# The Prognostic Role of Micro-RNAs in Head and Neck Cancers: An Umbrella Review

**DOI:** 10.3390/jpm11080821

**Published:** 2021-08-21

**Authors:** Marco Mariani, Carolina Castagna, Stefania Boccia, Roberta Pastorino

**Affiliations:** 1 Department of Life Sciences and Public Health, Section of Hygiene, Università Cattolica del Sacro Cuore, 00168 Rome, Italy; marco.mariani02@icatt.it (M.M.); stefania.boccia@unicatt.it (S.B.); 2 Department of Woman and Child Health and Public Health-Public Health Area, Fondazione Policlinico Universitario A. Gemelli IRCCS, 00168 Rome, Italy; roberta.pastorino@unicatt.it

**Keywords:** head and neck cancer, miRNAs, prognosis, survival, recurrence, relapse, systematic reviews

## Abstract

We conducted an umbrella review which synthetizes the findings of systematic reviews available in the literature that investigate the prognostic role of miRNAs as potential biomarkers in the field of tertiary prevention of head and neck Cancer (HNC). We selected systematic reviews in English related to HNC, with meta-analysis of observational studies that reported quantitative prognostic measures, hazard ratios (HRs), overall survival (OS) or disease-free survival (DFS). The methodological quality of the included reviews was assessed by using the AMSTAR-2 tool. The most reported miRNAs were the following: miRNA2, Let7 family and miR17, etc. Four out of six reviews particularly emphasized the link between miRNA21 expression and HNC patients. Recently the cumulative effects of sets of miRNAs have been increasingly studied and might be a stronger predictor of survival than single miRNA.

## 1. Introduction

Head and neck cancer (HNC) is a heterogeneous group of neoplasms which develop from different tissues such as oral and nasal cavities, paranasal sinuses, pharynx and larynx. It represents the sixth most common cancer and the seventh cause of cancer-related deaths worldwide [1]. HNC accounts for about 4% of all cancers in United States. An estimated 14.620 deaths (10.980 men and 3.640 women) from head and neck cancers occurred in 2019 in USA [2]. HNC can be related to environmental and lifestyle risk factors, including alcohol and hot drinks consumption, tobacco use, HPV (Human Papilloma Virus) or EBV (Epstein–Barr Virus) infection, poor oral and dental hygiene, malnutrition, gastroesophageal or laryngopharyngeal reflux disease and occupational exposure to chemicals and fumes [3]. The anatomy of head and neck region results in complicated patterns of tumor invasion and consequently in difficulties in treating patients suffering from these diseases [4]. The majority of patients present already advanced stages of cancer at diagnosis, characterized by local aggressiveness and high potential for local and systemic metastasis [5].

Due to HNCs’ high mortality and morbidity, support from the development of new biomarkers and personalized care for patients is urgently needed [1]. The role of micro-RNAs (miRNAs) as new epigenetic biomarkers aimed at improving early diagnosis, predicting prognosis and establishing effective cancer therapies has recently received considerable attention [6]. MiRNAs represent a class of highly conserved non-coding small molecules of RNAs (containing about 20–22 nucleotides) that regulate gene expression: clinical studies highlight that many miRNAs are involved in several biological processes, such as cellular proliferation, differentiation, migration, apoptosis, survival and morphogenesis [7]. Several studies were conducted in relation to miRNAs as biomarkers for cancer prognosis in many tumors, such as HNC. Even if the body of evidence in relation to miRNAs and HNC prognosis is growing, there is wide variability among studies regarding aims, miRNA measurement methods, tumor sites and different miRNAs among studies [1]; in fact, miRNA can be detected in both extracellular and intracellular environments. In addition, consistency between many miRNA signatures is lacking, and the differences may be due to source of miRNA, sample size and comorbidities of the studied samples [8]. Recently, in order to address the significance of miRNAs as potential prognosticators in HNC, some articles [1,9] studied the impact of miRNA by identifying the pooled effect size across all HNC prognostic studies, resulting in a summary estimates of the relationship between miRNAs expression and risk of death via pooling the hazard ratio across all selected studies with the aim of providing a better understanding of the survival outcome of HNC patients. Hence, the aim of this article was to conduct an umbrella review, i.e., a review of reviews, that compiles and summarizes all the evidence from existing reviews in order to investigate on the prognostic role of miRNAs as biomarkers in the field of tertiary prevention of HNC for the purposes of providing a high-level overview.

## 2. Materials and Methods

This umbrella review synthetizes the findings of reviews already available in the literature. The researchers systematically searched electronic databases for systematic reviews investigating the potential role of miRNAs as prognostic biomarkers in patients suffering from head and neck cancer.

### 2.1. Literature Search

An online search was conducted on the following searching engines: Pubmed, ISI Web of Science and Scopus. The search was limited to reviews published in the English language from their commencement until April 2021. We used the Population, Intervention, Comparator, Outcome and Study design (PICOS) model search strategy. Through the use of specific keywords, we constructed and launched the following search string: *“((prognosis OR prognostic OR survival OR recurrence OR relapse) AND “miRNA” AND “cancer” AND (“systematic review” OR “meta-analysis”))”.* Two reviewers (C.C. and M.M.), by using Mendeley software, independently screened titles and abstracts of all records identified. Full texts of all potentially eligible studies were obtained and assessed independently by two reviewers (C.C. and M.M.) against the eligibility criteria. At all levels, disagreements were resolved by discussion or by involving a third reviewer (R.P. or S.B.) when consensus could not be reached. We excluded studies if their full texts were not available. Using a standardized data extraction form, we independently extracted data from each study that were eligible for inclusion.

### 2.2. Eligibility Criteria

Articles were eligible if they were systematic reviews with meta-analysis of observational studies that reported quantitative prognostic measures, hazard ratios (HRs), overall survival (OS) or disease-free survival (DFS). Overall survival is a rate that represents the percentage of people in a study or treatment group who are still alive for a certain period of time after they were diagnosed with or started treatment for a disease, such as cancer. Disease-free survival in cancer represents the length of time, after primary treatment, the patient survives for without any signs or symptoms of that cancer. In a clinical trial, measuring the disease-free survival is one method to observe how well a new treatment works.

### 2.3. Quality Assessment

The methodological quality of the included reviews was assessed independently by two authors in duplicate and disagreements were resolved by consensus or involving a third author (R.P. or S.B.). The quality assessment tool used was the Assessment of Multiple Systematic Reviews (AMSTAR-2) [10], which consists of 16 domains presented in the form of questions. The possible answers to these questions include ‘Yes’ if it denotes a positive result; the article presents a weakness if the answerer is negative ‘No’ (or it cannot be provided) or ‘Partial Yes’ in case of partial adherence to the standard. The AMSTAR overall judgment was based on the assessment of specific critical domains such as the following: (I) presence of a registered protocol before the commencement of the review; (II) evaluation of the risk of bias of the studies included; (III) appropriateness of meta-analytic methods—if applicable; (IV) consideration of risk of bias when interpreting the results of the review; and (V) assessment of presence and likely impact of publication bias. After grading each study, a judgement of the overall quality (high, moderate, low and critically low) was made by taking into account the relative importance of potential sources of bias.

### 2.4. Data Extraction

The following data were extracted independently by two authors (C.C. and M.M.) from the following included studies: name of the first author, publication year, number of qualitative studies, number of patients included in the review, miRNAs studied, level of regulation of miRNAs, primary study OS and DFS reported in the qualitative synthesis and OS (HR) and DFS (HR) of the quantitative synthesis. Results reporting a *p*-value < 0.05 were considered statistically significant.

## 3. Result

### 3.1. Study Selection 

**Figure 1:**
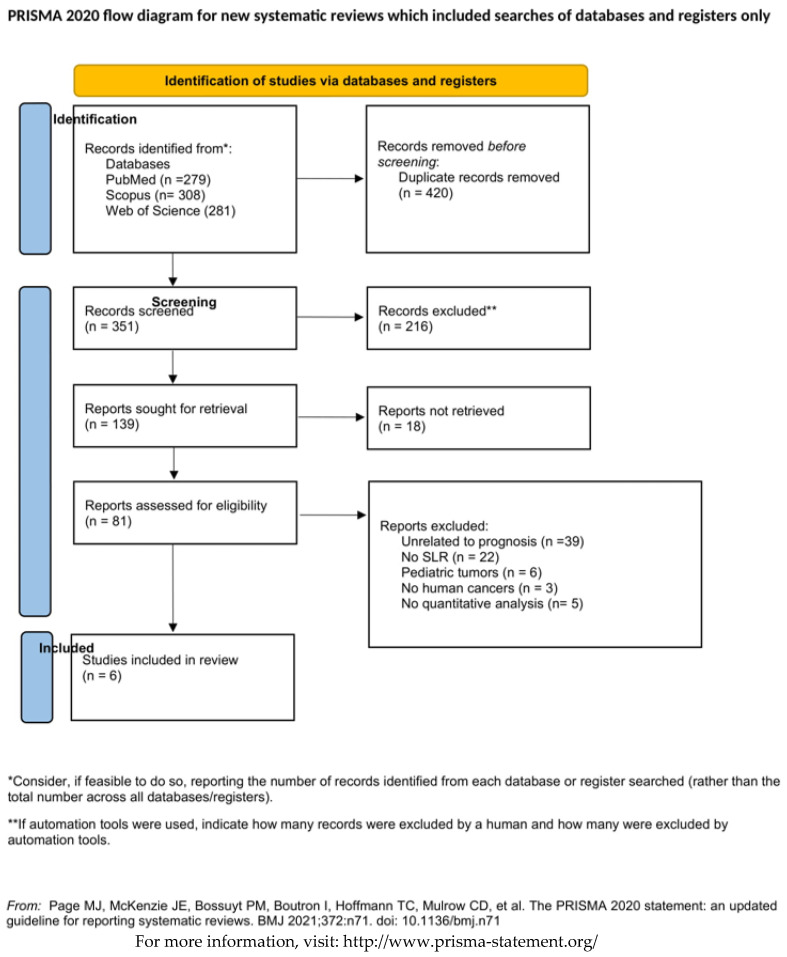
Flowchart of the literature research by database. SLR systematic literature review.

Across all databases, our search identified 662 potentially relevant papers. After removal of duplicates, 351 papers were left. As described in Figure 1, 216 articles were excluded because they did not meet the inclusion criteria after title and abstract reading. A total number of 135 full manuscripts were assessed for eligibility, which resulted in the inclusion of 77 eligible papers. Of these, 71 articles were excluded for the following reasons: (I) unrelated to prognosis or tertiary prevention 50.6% (n. 39); (II) no systematic reviews 28.6% (n. 22); (III) articles regarding pediatric tumors 7.8% (n. 6); (IV) unrelated to human cancers 3.9% (n. 3); (V) articles did not present quantitative analysis 3.9% (n. 3); and (VI) full text not available 5.2% (n. 4). Eventually, a total of six systematic reviews were included in this umbrella review.

### 3.2. Results of the Quality Assessment

As shown in Table 1, according to the qualitative assessment tool, the AMSTAR-2, one out of the six systematic reviews (16.7%) was evaluated as ‘critically low’ [11] because it showed more than one weakness among the critical domains previously described. Specifically, this review presented negative answers with respect to two critical questions related to the adequate investigation of publication bias (small study bias) and discussion of the impact on the results of the review and the RoB in individual studies when the results of the review were interpreted/discussed. Four included studies (66.6%) were evaluated as ‘low’ quality [9,12,13,14] since they presented only one critical flaw. All of them [9,12,13,14] did not satisfy the risk of bias in individual studies when the results of the review were interpreted/discussed (i.e., whether a satisfactory technique for assessing the risk of bias in individual studies that were included in the review was used). Eventually, a review (16.7%) [1] resulted as qualitatively ‘moderate’: no critical flaws were assessed, but it had more than one weakness related to an adequate description of the included studies in detail and the sources of funding for the studies included in the review.

### 3.3. Main Findings

The total population of the individuals included in the meta-analysis varied from 422 to 8194 individuals. The most reported and studied miRNAs included the following: miRNA21 from all of the included studies; the Let7 family (c, d and g), miRNA17, 18 family (a and b), 20a, 29 family (a, b, c), 125b, 375 and 451 by three reviews (50%); miRNA34a, 155, 181, 205, 210, 218 and 363 by two reviews (33%); and other miRNAs were reported by a single review. Two studies [1,9] performed a meta-analysis, pooling HRs (95% CIs) from different miRNAs to obtain an overall estimate of the effect of the combination of more miRNAs. One study [14] reported that OS from upregulated miRNAs (from 25 primary studies) possess a pooled HR 1.76 (95% CI 1.43–2.17) and downregulated ones (from 20 primary studies) possess a pooled HR 2.02 (95% CI 1.43–2.17). The other study [1] reports OS, by taking together upregulated and downregulated, included miRNAs that show an overall HR of 1.20 (95% CI: 0.89–1.60) and, when stratified by level of regulation, upregulated miRNAs have a pooled HR 4.64 (95% CI, 1.05–2.58), while downregulated ones have a pooled HR 0.94 (95% CI 0.65–1.39). For what concerns the DFS in this latter study [1], when upregulated and downregulated miRNAs were taken together, they showed an overall HR of 2.60 (95% CI: 1.91–3.51); in particular, upregulated miRNAs only (from 10 studies) showed a pooled HR 2.64 (95% CI 1.93–3.62), while downregulated ones have a pooled HR 2.10 (95% CI 0.71–6.20). A total of four reviews [1,11,12,13] assessed miRNA21 expression in HNC patients. The pooled HRs from the OS analysis ranged from 1.46 to 1.81 and all were statistically significant. Among these four, two [1,13] showed that the expression of miRNA21 is not significantly associated with a lower DFS probability. The main findings of the systematic reviews and meta-analysis are summarized in the Table 2 below.

## 4. Discussion

### 4.1. Summary of the Findings

The aim of this review was to summarize evidence relative to the potential prognostic role of miRNAs as biomarkers in HNC. Some miRNAs were demonstrated to have tumor-suppressing and oncogenic roles according to their level of expression (upregulation or downregulation) in HNC patients [11]. In this umbrella review, a focus was placed on their role in prognosis; therefore, OS and DFS of patients with different miRNAs (assessed together and/or individually) levels of expression were evaluated. Among all the reviews, we found a recurring element, i.e., that the most frequently studied miRNA was miRNA21, which was reported either in the OS or DFS statistical analyses. The OS analysis showed a significantly lower prognosis when miRNA21 (individually or in combination with other miRNAs) was upregulated. In particular, miRNA21 has been associated with different cancer types (in both solid and hematological tumors). Indeed, it was found to be overexpressed in different kinds of tumors, such as glioblastoma, breast, lung, colon, pancreas, prostate, stomach cancer, hepatocellular carcinoma, ovarian cancer, cervical carcinoma, thyroid carcinoma and leukemia, apart from HNC.

As stated by some recent studies [15,16], miRNA-21, due to its large involvement in different pathways in both neoplastic and non-neoplastic diseases, cannot be considered a specific or reliable biomarker for HNC prognosis. However, in most reviews, even if the pooled HR was significant in most statistical analyses, its utility as a prognostic factor based on the relative strength can be considered moderate or weak as it does not reach a HR > 2 [17]. In order to increase the prediction of the prognosis, researchers have recently started to focus on the analysis of groups of different miRNAs together. Some studies [1,9,11,13,14] described specific sub-sets of miRNAs that were pooled together in order to better comprehend their association with HNC, i.e., by aggregating data from different studies. This process might produce more robust estimates [14] with respect to single studies and may increase statistical power, even if it does not permit a comparison among components of the panel, i.e., these panels of microRNAs as a whole are predictive of poor prognosis in HNC patients [14]. In fact, regarding the OS, two reviews (Kumarasamy et al., 2019; Sabarimurugan et al., 2018) [1,9] reported a meta-analysis where HRs from different miRNAs expressions were pooled together. One showing that downregulated miRNAs, when taken together, have a pooled HR 2.02 (95% CI 1.43–2.17) (Sabarimurugan et al., 2018) [9] and the second one (Kumarasamy et al., 2019) [1] showing that upregulated miRNAs, when taken together, have a pooled HR 4.64 (95% CI, 1.05–2.58). Furthermore, this latter study (Kumarasamy et al., 2019) [1] highlights that a higher predictive power is obtained in the meta-analysis of DFS of different miRNAs that results in a pooled HR of 2.64 (95% CI 1.93–3.62) and of 2.14 (95% CI 0.73–6.18), respectively, for upregulated and downregulated miRNAs.

### 4.2. Strengths and Limitations

Overall, many faints can occur in this kind of study, as reported in literature. First, conclusions relative to the level expression (upregulation/downregulation) can be misleading as thresholds regarding the cut-off definition are not unequivocally set, and the results from primary studies more widely correspond to mean or median values of the laboratory that performs the analysis. In addition, the levels of expression of miRNAs depend on the tissues that are analyzed (plasma, serum and tumor tissue) as miRNAs have widely variable levels of expression according to the cell type [18]. Other problems in relation to miRNAs can occur, and they range from the challenging techniques of the measurement of miRNAs to the duration of the follow-up of patients and characteristics of the sample population (age, ethnicity, tumor stage and tumor type) [12,18].

Another limitation to consider is that we did not conduct a specific meta-analysis stratification in accordance with the site of cancer occurrence. In fact, comprehensive meta-analyses including all cancer sites of occurrence were pooled together in the included reviews. This requires further investigation since the prognosis and the overall survival of head and neck cancer significantly differ according to this factor. Another issue that is highlighted by the studies included in this umbrella review is the scarce specificity related to the pathology or tumor site of miRNAs. In particular, even though miR-21 is well known for its prognostic potential in solid cancers, it is not specific to detecting HNC and is, therefore, not a particularly ideal miRNA for HNC prognosis [1].

## 5. Conclusions

In conclusion, to the best of our knowledge, this study represents the first attempt to summarize published reviews regarding the prognostic role of miRNAs in head and neck cancer. Recently, the cumulative effects of sets of miRNAs together have been increasingly studied, and they might be stronger predictor of survival than single miRNAs. Eventually, different issues arise from the analysis of miRNAs, and the above-mentioned problems still need to be addressed by performing large scale studies in order to verify and enhance the power of evidence and clinical utility of these biomarkers both individually and in combination.

## Figures and Tables

**Table 1:** Quality Assessment of the included reviews using the AMSTAR-2 checklist. Items with * represent the critical domains.

Review	Item																Overall Judgement
	1. Question and Inclusion	2. Protocol *	3. Study Design	4. Comprehensive Search *	5. Study Selection	6. Data Extraction	7. Excluded Study Justification	8. Included Studies Details	9. Risk of Bias *	10. Funding Sources	11. Statistical Methods *	12. Rob on Methanalysis	13. Rob in Individual Studies *	14. Explanation for Hetherogenity	15. Publication Bias *	16. Conflict of Interest	
Fu 2011	Yes	Partial Yes	Yes	Yes	Yes	No	Partial Yes	Partial Yes	No	Yes	No	Yes	No	Yes	Yes	Yes	LOW
Jamali 2015	Yes	Partial Yes	Yes	Yes	Yes	Yes	Partial Yes	Partial Yes	Yes	No	Yes	No	No	Yes	Yes	Yes	LOW
Lubov 2017	Yes	Partial Yes	Yes	Yes	Yes	No	Partial Yes	Yes	Yes	No	Yes	No	No	Yes	No	Yes	CRITICALLY LOW
Troiano 2018	Yes	Partial Yes	Yes	Yes	Yes	Yes	Partial Yes	Yes	Yes	No	Yes	Yes	Yes	Yes	No	Yes	LOW
Kumarasamy 2019	Yes	PartialYes	Yes	Yes	Yes	Yes	Partial Yes	No	Yes	No	Yes	Yes	Yes	Yes	Yes	Yes	MODERATE
Sabarimurugan 2019	Yes	Yes	Yes	Yes	Yes	Yes	Partial Yes	Yes	Yes	Yes	Yes	No	No	Yes	Yes	Yes	LOW

**Table 2:** Characteristics and main findings of the six systematic reviews and meta-analysis included.

Author	Year	Number of Qualitatative Studies	Number of Patients	miR		Qualitative Studies		Quantitative Studies	
					Level of Regulation	OS	DFS	OS HR (95% CI)	DFS HR (95% CI)
Fu X	2011	4	422	21	U	− *		1.46 (1.13–1.87)	
Jamali Z	2015	25	2006	17, 20a, 153, 200c, 203, 375, 451, Let-7g	D	− *			
				193b, 205	D		− *		
				126a	D		−		
				18a, 19a, 20a, 21	U	− *			
				134a, 201	U		− *		
				155	U	−		1.57 (1.22–2.02)	1.00 (0.42–2.6)
Lubov J	2017	116	8194	17, 20c, 21a, 26a, 195, 203, 218, 375, Lin28B	D	− *			
				34a, 34c-5p, 126a, Let7d, Let-7g	D		− *		
				205, 451	D	− *	− *		
				9, 18a, 19a, 20a, 23a, 155, 206, 210, 1246	U	− *			
				21	U	− *		1.81 (0.66–2.95)	
				130b-3p, 134, 196a, 213p, 372, 373, 965p, 1413p	U		− *		
Troiano G	2018	15	1200	16	D		−	1.95 (1.28–2.98)	
				17	D	− *		2.65 (2.07–3.3	
				20a, 32, 204	D	−			
				101, 125	D	− *			
				21, 155-5p, 196a, 372, 373, 455-5p	U	−			
				29b, 181a, 181b, 1246	U	− *			
Kumarasamy C	2019	50	6867	34a	D				0.19 (0.01–130.51)
				34c-5p	D			4.36 (2.38–8.00)	
				200b	D			1.19 (0.66–2.18)	
				**let-7g, 17, 20a, 26a, 29c, 34c-5p, 142, 146a, 155, 195, 200b, 203, 212, 218, 300, 375, 451, 548b**	D			2.02 (1.42–2.86)	
				18a	U			1.87 (1.05–3.33)	
				21	U			1.59 (1.15–2.19)	1.47 (0.81–0.27)
				125b	U			2.3 (0.40–13.40)	
				**let7a, 9, 18a, 19a, 20a, 21, 93, 100, 125b, 155, 206, 375, 377-3p, 483-5p, 1246**	U			1.76 (1.43–2.17)	
				**34, 126a, 205**	D				2.10 (0.72–6.17)
				**21, 21-3p, 96-5p, 130b-3p, 134, 141-3p, 210, 372, 373**	U				2.64 (1.92–3.66)
				**34, 126a, 205;** **21, 21-3p, 96-5p, 130b-3p, 134, 141-3p, 210, 372, 373**	D and U				2.60 (1.91–3.51)
Sabarimurugan S	2019	21	5069	92b	D	+			
				**18b, 184, 324-3p, 3188**	D	+ *			
				**29c, 103, 204, 451, 483-5p,744**	D	− *			
				**18b, 29c, 92b, 103, 184, 204, 324-3p, 451, 483-5p, 744, 3188,**	D			0.95 (0.65–1.39)	
				663, let-7c	U	−			
				**19b-3p, 18a, 29a, 92a**	U	+ *			
				**10b,17-5p, 21, 22, 572, 638, 1234**	U	− *			
				**18b, 29c, 92b, 103, 184, 204, 324-3p, 451, 483-5p, 744, 3188**	D			0.95 (0.65–1.39)	
				**let-7c, 10b, 17-5p, 18a, 19b-3p, 21, 22, 29a, 92a, 572, 638, 663, 1234,**	U			1.64 (1.05–2.58)	
				**18b, 29c, 92b, 103, 184, 204, 324-3p, 451, 483-5p, 744, 3188; let-7c, 10b, 17-5p, 18a, 19b-3p, 21, 22, 29a, 92a, 572, 638, 663, 1234,**	D and U			1.19 (0.89–1.60)	

*U*—miR is upregulated; *D*—miR is downregulated; − or *+* represent reduced or increased, respectively; OS or DFS according to the column; * = statistically significant; in bold, different miRs HRs (95% CI) were pooled together in a meta-analysis, performed to obtain an overall estimate of their effect when combined. Non-bold miRs are those that, in separate analysis by type of miR, showed the same level of regulation, association and significance in the OS or DFS.

## Data Availability

Not applicable.
